# Structural Dynamics Analysis of USP14 Activation by AKT-Mediated Phosphorylation

**DOI:** 10.3390/cells13110955

**Published:** 2024-05-31

**Authors:** Raju Dash, Non-Nuoc Tran, Sung Bae Lee, Byung-Hoon Lee

**Affiliations:** 1 Department of New Biology, Daegu Gyeongbuk Institute of Science and Technology (DGIST), Daegu 42988, Republic of Korea; dash_raju@dgist.ac.kr (R.D.); tnnuoc_88@dgist.ac.kr (N.-N.T.); 2 Department of Brain Sciences, Daegu Gyeongbuk Institute of Science and Technology (DGIST), Daegu 42988, Republic of Korea

**Keywords:** USP14, phosphorylation, AKT, blocking loop, ubiquitin-proteasome system, molecular dynamics simulation

## Abstract

Ubiquitin-specific protease 14 (USP14), one of the three major proteasome-associated deubiquitinating enzymes (DUBs), is known to be activated by the AKT-mediated phosphorylation at Ser432. Thereby, AKT can regulate global protein degradation by controlling the ubiquitin-proteasome system (UPS). However, the exact molecular mechanism of USP14 activation by AKT phosphorylation at the atomic level remains unknown. By performing the molecular dynamics (MD) simulation of the USP14 catalytic domain at three different states (inactive, active, and USP14-ubiquitin complex), we characterized the change in structural dynamics by phosphorylation. We observed that the Ser432 phosphorylation induced substantial conformational changes of USP14 in the blocking loop (BL) region to fold it from an open loop into a β-sheet, which is critical for USP14 activation. Furthermore, phosphorylation also increased the frequency of critical hydrogen bonding and salt bridge interactions between USP14 and ubiquitin, which is essential for DUB activity. Structural dynamics insights from this study pinpoint the important local conformational landscape of USP14 by the phosphorylation event, which would be critical for understanding USP14-mediated proteasome regulation and designing future therapeutics.

## 1. Introduction

The dynamic regulation of a balanced and functional cellular proteome is known as proteostasis, which involves the control of the synthesis, folding, trafficking, and clearance of proteins [1,2]. Proteostasis is essential for cell function and survival, and its dysregulation results in various diseases, including cancers and neurodegenerative diseases [3]. The maintenance of proteostasis is highly dependent on the turnover rate of protein substrates, which is primarily regulated by the ubiquitin-proteasome system (UPS). In UPS, the 26S proteasome (2.5–3 MDa) can degrade at least 80% of intracellular proteins by recognizing the ubiquitin (Ub) tag as a degradation signal [4,5]. Also, the degradation process on the proteasome can be tightly regulated by a series of Ub-dependent mechanisms, including Ub chain recognition (Ub receptors), elongation (E3 Ub ligase), and cleavage of the Ub chain (deubiquitinating enzymes, DUBs) [5,6]. The activity of the proteasome is controlled dynamically by a number of proteins, which are reversibly associated with it [4,7]. As an example, Ub-specific protease 14 (USP14, or Ubp6 in budding yeast) can stabilize protein substrates against degradation by eliminating Ub chains from substrates bound to the proteasome [8,9], while inducing proteasomal ATPase activity and gate opening [10,11,12,13].

**Figure 1:**
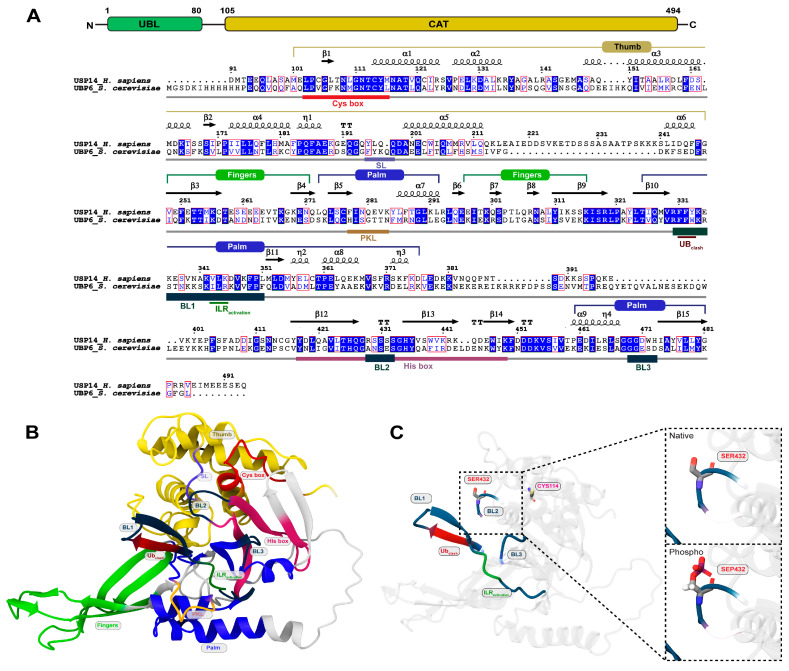
The sequence mapping and structure of USP14 with its domain organization and phosphorylation site. (**A**) USP14 domain mapping and pairwise sequence alignment of the human USP14 catalytic domain and its yeast homolog, Ubp6. Marked regions at the top of the sequence alignment represent common USP subdomains, including thumb, fingers, and palm. The marked segment at the bottom of the alignment highlights different functional elements in the USP14 catalytic domain. Conserved residues are shaded in blue, while residues in the red boxes represent similarity across groups. (**B**) Cartoon rendering of the cryo-EM structure of the proteasome-bound USP14 catalytic domain (i.e., active USP14, retrieved from PDB ID: 7W3H, chain ID: x) showing the different subdomains and structural elements of USP14 critical for catalytic activity and proteasome association. (**C**) Structure of the USP14 catalytic domain showing the position of the phosphorylation site (Ser432), blocking loops (BL1, BL2, and BL3), and catalytic cysteine in the active state of USP14.

USP14 is one of the most studied DUBs because of its capability of proteasome association and regulation and because it is targeted for therapeutic intervention in diverse diseases such as neurodegeneration, inflammation, and cancer [8,14,15,16,17]. USP14 belongs to the cysteine protease DUB class and contains a catalytic USP domain at its C-terminus and a ubiquitin-like (UBL) domain at the N-terminus (Figure 1A) [18]. Like other DUBs in the USP family, the catalytic domain of USP14 contains a Ub-binding cleft for Ub cleavage formed by three common subdomains of thumb, palm, and finger regions (Figure 1A,B). The binding of Ubp6/USP14 to Ub and the proteasome is mediated by several blocking loops of the catalytic domain known as BL1 (residues 330 to 351), BL2 (residues 429 to 433), and BL3 (residues 469 to 473), where some of these loops maintain the interaction with the OB domains of the ATPase RPT ring of the proteasome (Figure 1A,B) [13,19,20]. Notably, in the free form of USP14, BL1 and BL2 obstruct the active site, making it implausible for the C-terminus of Ub to enter the active site, explaining why its free form displays only sluggish deubiquitinating activity [18,21]. On the other hand, in proteasome-bound states, the positions of BL1 and BL2 are displaced by the interaction with the OB ring, allowing USP14 cleavage activity to increase up to 800-fold [8,19]. A recent cryo-EM-based study revealed that BL1 and BL2, together with a switching loop (SL), confer an inhibitory network of blocking loops, where BL1 directly interacts with BL2, while BL2, with SL, all of which are responsible for clashing with the Ub moiety in a free form of Ubp6/USP14 [19].

Interestingly, in the absence of a proteasome, USP14 can also be activated by post-translational modification, i.e., phosphorylation of the Ser432 residue in the BL2 loop by the AKT kinase (Figure 1C), by which AKT can regulate UPS-mediated global protein degradation [22]. It was reasoned that phosphorylation at this site may induce conformational changes in BL2 because of the repulsive effect of phosphorylation in the negatively charged patch, which may allow the BL2 loop to move away from its inhibitory conformation. However, the mechanistic details at the atomic level remain elusive. For example, some questions still need to be addressed: how phosphorylation induces conformational changes in the inhibitory network of blocking loops at a molecular level, the effect on overall structural dynamics and the active site groove, and the structural impact on Ub binding. To address these questions, we compared the dynamics and behavior of the USP14 catalytic domain between its native and phosphorylated states in three different structural conformations (inactive, active, and USP14-Ub complex) by using molecular dynamics (MD) simulation. Our study discloses a novel mechanism for phosphorylation-induced activation of USP14, which is associated with the conformational changes of the enzyme in the inhibitory network of blocking loops as well as the dynamic change of its electrostatic interactions with Ub.

## 2. Materials and Methods

### 2.1. Model Building

For the simulation study, the crystal structure of human USP14 was obtained from the Protein Data Bank; PDB id: 2AYN [18] was considered a free form of USP14 (inactive). The structure was prepared in the Schrödinger suite, Ver 2022-4 (Schrödinger, LLC, New York, NY, USA) [23] by revising residual bond orders and charges, deleting water molecules, and adding hydrogen atoms. The structure has missing residues from Leu140-Met146 and Ser222-Lys239, which were added and fixed by the pdbfixer utility of the OpenMM package in loop conformation [24]. The protonation state of amino acids, including glutamic acid, aspartic acid, and histidines, was fixed at a neutral pH. In order to optimize the corrected structure, a restrained minimization was additionally applied using Optimized Potentials for Liquid Simulation (OPLS4) force field with an RMSD of the protein-heavy atom changed to 0.30 Å. Additional loop refining was also incorporated to refine newly generated missing loops using the refine loops utility of the Schrödinger suite Ver 2022-4, using the VSGB solvation model and OPLS4 field.

The active form of USP14 (proteasome-bound) was constructed using the cryo-EM model of USP14-bound proteasome structure (PDB id: 7W3H, chain: x [13]). To maintain consistency with the inactive USP14, only the catalytic part was kept and used for the simulation. The Ub-bound form of USP14 was also prepared from the USP14-bound proteasome structure (PDB id: 7W3H, chains: x, y) [13], keeping the amino acid length for the catalytic domain the same as the other two models. Phosphorylation at Ser432 was introduced using the structure builder tool of Molecular Operating Environment (MOE) software, Ver 2015 (Chemical Computing Group ULC, Montreal, QC, Canada), by following an energy minimization process and applying the Amber12:EHT force field [25] with the reaction field solvation model. The resultant structure was used for further analysis. All structures were prepared for simulation following the same approach described above for inactive USP14, except for loop refinement.

### 2.2. Molecular Dynamics (MD) Simulation

MD simulations were carried out using the academic version of Desmond (D E Shaw Research (DESRES)) software, Ver 2023-4, using the OPLS4 force field [26], as described earlier [27,28,29]. Using the explicit solvation model (Monte Carlo simulated transferable intermolecular potential 3 points (TIP3P) water model [30]), all structures were solvated in an orthorhombic box with an extension of 10 Å in each direction. These structures included the free form of USP14 (inactive), the proteasome-bound form of USP14 (active), and the USP14-Ub complex. To neutralize the systems, additional counterions (Na^+^/Cl^−^) were added to the systems, and the salt concentration of the system was adjusted to 0.15 M to maintain physiological conditions. Each solvated system was subjected to minimization and equilibration processes before the MD simulations using the default Desmond protocol comprised of a series of minimization (restrained) and MD, as described earlier [29,31]. The simulation was performed under thermodynamical conditions, and the isotropic Martyna–Tobias–Klein barostat [32] and the Nose–Hoover thermostat [33] were used to maintain the pressure at 1 atm and temperature at 300 K, respectively. The long-range electrostatic interactions were calculated using the particle mesh Ewald method [34], and the short-range electrostatic interactions were analyzed using a cut-off of 9.0 Å. For bonded and non-bonded interactions within the short-range cut-off, the multistep RESPA integrator was used to integrate the equations of motion with an inner time step of 2.0 fs [35]. An outer time step of 6.0 fs was utilized for non-bonded interactions beyond the cut-off. Finally, using the NPT ensemble method, each simulation was produced at the indicated time for each replica (Table S1), and the coordinates were saved with an interval of 100 ps.

Different parameters for trajectory characterization, such as root mean square deviation (RMSD), root mean square fluctuation (RMSF), and radius of gyration (Rg), were used to characterize the resulted trajectory data by using the default Python script provided by Desmond (D E Shaw Research (DESRES)) software, Ver 2023-4. MDAnalysis (https://github.com/MDAnalysis/) was utilized to generate a representative PDB for RMSF analysis [36]. Define Secondary Structure of Proteins (DSSP) was used to calculate the occupancy of secondary structure elements (SSE) [37]. In addition, principal component analysis (PCA) was performed using the Bio3D packages, Ver 2.4-4 of R [38]. Furthermore, to find sets of modes that are comparable to each other based on normal modes or principle components (PCs), root mean square inner products (RMSIPs) were computed using the first 20 PCs. The RMSIP values were calculated using Bio3D [38], ranging from 0 to 1, with 0 indicating orthogonality and 1 indicating equal directionalities of sample subspaces. PyMOL 2.4.0 (The PyMOL Molecular Graphics System, Ver 2.4.0 Schrödinger, LLC) was used for structural visualization.

### 2.3. Trajectory Clustering Analysis

In order to group the complex structure from the large MD simulation trajectory, we used the Desmond trajectory clustering analysis tool by considering the backbone RMSD matrix of the BL2 loop for the active and inactive states and the backbone RMSD for the USP14-Ub complex. Clustering was done in up to 20 clusters with a frequency of 5.

### 2.4. Free Energy Landscape (FEL)

Using the InfleCS clustering approach based on the Gaussian Mixture Model (GMM), we map the subspace conformation-free energy surfaces of the trajectory. During calculation, the number of grids 80 was used with a minimum and maximum number of Gaussian components, 2 and 10, respectively, which are deemed to fit densities for each GMM. A maximum of 1000 iterations were taken into consideration to obtain GMM functions. Structural characteristics such as Rg and RMSD were considered structural determinants for computing free energy surfaces [39,40].

## 3. Results

In order to understand the conformational dynamics of the USP14 catalytic domain changes upon Ser432 phosphorylation, we performed MD simulation at a microsecond timescale. For a detailed mechanistic insight, phosphoserine (SEP) was introduced at the 432 position in three different conformational states of the USP14 catalytic domain as previously reported [13,18], that is, the free form of USP14 (inactive), the proteasome-bound form of USP14 (active), and the Ub-bound form of USP14 (USP14-Ub complex), and a simulation was performed along with their respective native conformers. For each condition, the simulation trajectory was collected and analyzed at least from four independent simulations, where the simulation length of each replica and corresponding RMSDs are shown in Table S1 and Figures S1–S3, respectively. The RMSDs, which are calculated for every conformation in relation to the identical initial conformation, showed a similar trend for each replica for a specific system, indicating that the obtained data is reproducible and can be used for further analysis.

### 3.1. Active USP14 Displays Different Dynamics from Its Inactive state

**Figure 2:**
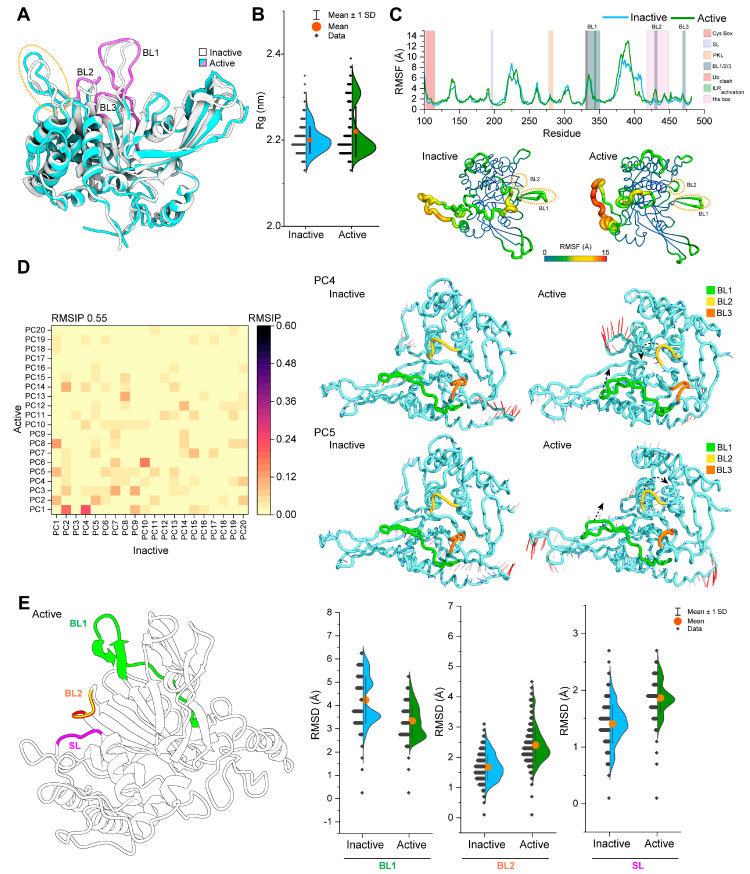
Differential conformational dynamics between inactive and active USP14. (**A**) The overlay crystal structure of inactive and active USP14 shows the conformational difference between the two states in blocking loops and an additional region highlighted by the dotted orange circle. (**B**) The half-violin plot shows the difference in distribution and the mean value of the calculated radius of gyration (Rg) between the inactive and active USP14. The orange circle, error bars, and black dots in the half-violin plot indicate the average, standard deviation (SD), and each Rg value, respectively. (**C**) Changes in the residual fluctuation were measured utilizing root mean square fluctuation (RMSF) by considering the c-alpha atom of the starting structure of the simulation for both inactive and active types and represented as a line plot. Colored, shaded boxes indicate different regions of functional elements. At the bottom, the differences between the residual fluctuations of inactive and active states are highlighted by rainbow color-coded tube representations, where a red-shaded wide tube indicates the region with high RMSF fluctuations. In contrast, the narrow tube with a blue shade indicates the region has low flexibility. The dotted circles highlight the regions of BL1 and BL2, describing the changes in RMSF during the simulation. (**D**) A color-coded heat map showing the overlap of the RMSIP matrix was analyzed for the first 20 principal components (PCs) of the inactive and active USP14 structures. On the right side, the opening and closing motions of BL1 and BL2 of active USP14, which were traced in PC4 and PC5 through PCA analysis of MD trajectories of inactive and active states, are shown by porcupine plots, where the magnitude and direction of dynamics are represented by the length and direction of mod vectors. The black arrow indicates the direction of opening or closing motion, where the color scale ranging from red to blue in the mod vectors indicates atomic displacements from high to low. (**E**) Structural dynamics of blocking loops (BL1 and BL2) and SL in inactive and active USP14 simulations are measured by RMSD calculation of c-alpha atoms and shown in a half-violin plot (right side) along with their 3D structure representation (left side), which was constructed based on active USP14 structure. The orange circle, error bars, and black dots in the half-violin plot indicate the average, standard deviation (SD), and each RMSD value, respectively.

Prior to analyzing the effects of phosphorylation in the USP14 catalytic domain, it is essential to reveal the fundamental differences in conformational dynamics between inactive and active states by MD simulation. 3D structural studies highlighted substantial conformational changes in BL1 and BL2 [21], the latter being rotated into 90° and moved 4 Å away to adopt Ub in the active site groove. In addition to conformational changes of BL1 and BL2, an additional conformational change was also observed in the region from Phe183 to Asp199 of USP14 (Figure 2A), including the SL element, which is known to be displaced with the BL2 loop for Ub entry in the catalytic site [19].

When comparing simulation trajectories, Rg analysis between the inactive and active states revealed that the active structure has a more extensive distribution in Rg than the inactive state, indicating that the active state is more dynamic (Figure 2B). Specifically, the region of residue 370 to 415 (spanning from the palm region) in the active state was found to be more flexible in the simulation than the inactive structure, as represented by RMSF analysis (Figure 2C). A substantial difference in RMSF was also observed in the BL2 loop but not in the SL region between inactive and active states. Moreover, conformational changes in BL1 were not apparent during the simulation, but some minor changes occurred in the Cys box region in the active structure. Whether the overall dynamic motion of the catalytic domain is different between the active and inactive states was further analyzed using PCA analysis, which depicts the collective motions of the localized fluctuations through PCs. Consistent with Rg and RMSF analysis, the comparison of the top 20 PCs between inactive and active states by RMSIP calculations showed that dynamic motions are distinguishable between the two states (Figure 2D). Most of the differences in protein conformation were captured by PC1, followed by PC2 (Figure S4A,B), but these PCs failed to show the conformational flexibility in the BL2 region, which was also the case for PC3. However, when analyzing PC4 and PC5, substantial fluctuations are observed predominantly in the BL2 region, accompanied by SL shifting (Figure S5). Therefore, PC4 and PC5 were subjected to visualizing structural movements by porcupine plot, where PC4 represents the BL2 moving into the active site groove and PC5 shows the BL2 loop moving away (Figure 2D). In both PC4 and PC5, the active conformer shows substantial differences in the direction of the movement of residues 362 to 406 compared to the inactive conformer. PC5 in the active state also exhibited marked fluctuations in the region from 256 to 265 and 296 to 313, respectively, which are absent in the inactive conformer.

Next, we measured the conformational states of BL1, BL2, and SL during the simulation using RMSD calculations, as shown in Figure 2E. As expected, the conformational flexibility of BL2 in the active state was substantially higher than in the inactive state, and this trend is also similar for SL. However, the magnitude of conformational flexibility for BL1 was noticeably reduced for the active state, which was higher in inactive conformers.

### 3.2. Phosphorylation Changes the Conformational Dynamics of Blocking Loops in the USP14

**Figure 3:**
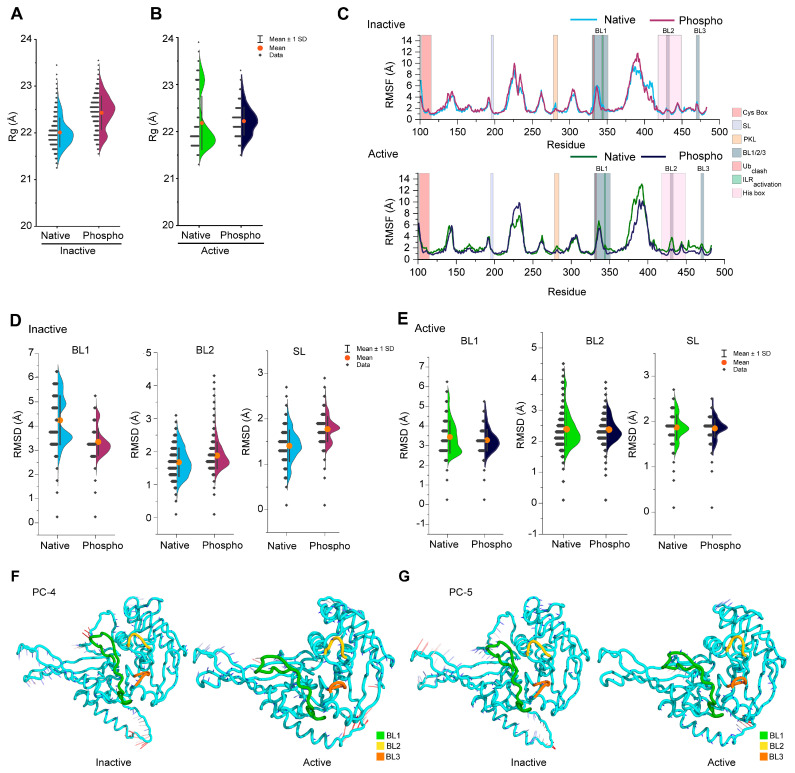
Effect of Ser432 phosphorylation on the conformational dynamics of USP14. Changes in overall conformational dynamics of USP14 in both native and phosphorylated forms were analyzed by radius of gyration (Rg), which were compared and shown in half-violin plots for both (**A**) inactive and (**B**) active states. (**C**) RMSF analysis in line plots shows the changes in residual fluctuation between the native and phosphorylated USP14 for both inactive (upper plot) and active (bottom plot) states. (**D**,**E**) Conformational changes in the inhibitory network of blocking loops (BL1, BL2, and SL) by phosphorylation in the inactive and active states were highlighted by RMSD analysis shown in half-violin plots, respectively. In all half-violin plots (including **A**,**B**,**D**,**E**), each single data point, the standard deviation (SD), and the average are shown by the black dot, error bars, and orange circle, respectively. (**F**,**G**) The porcupine plots highlight the contributions of PC4 and PC5 to phospho-inactive and active USP14, showing the differences in directed motion in the inhibitory network of blocking loops. The degree of atomic displacement from high to low is expressed by each color of mod vectors from red to blue, respectively.

Using the similar assessment parameters described in Figure 2, we next analyze the effect of phosphorylation on the conformational dynamics of the USP14 catalytic domain in both active and inactive states. Similar to Figure 2, the overall conformation of inactive USP14 was dramatically changed by phosphorylation, but the changes in the active state were not substantially noticeable compared to the inactive state (Figure 3A,B). However, no significant conformational changes were detected in the RMSF analysis, except in the regions ranging from residues 365 to 400. Notably, the BL2 region in the active state only showed a response to phosphorylation, where the loop seems to be stabilized by phosphorylation (Figure 3C). In addition, phosphorylation in the active USP14 also increased residual flexibility between residues 223–241, which was not seen substantially in the inactive USP14.

Next, we analyzed the changes in the conformational dynamics of the blocking loop network by phosphorylation for both inactive and active states. As shown in Figure 3D, phosphorylation reduced the conformational dynamics of the inactive USP14 BL1 loop but increased in the case of BL2 and SL (Figure 3D), a similar trend observed in Figure 2E. Active USP14 showed slightly reduced BL1 conformational flexibility due to phosphorylation, but no substantial differences were observed in the BL2 and SL loops (Figure 3E). We further analyzed the dynamic motion using PCA analysis to check whether the phosphorylation-induced relative changes in the loop dynamics are associated with the opening or closing motion. Unlike the comparative PCA analysis described in Figure 2 and Figure S5, the PCA of phospho-inactive USP14 failed to retrieve any remarkable dynamic motion at the BL2 loop, even in the first five modes (PC1–PC5), suggesting that phosphorylation did not induce much conformational transition of BL2 in the inactive state (Figure S6). On the contrary, phosphorylation either reduced (PC1, PC3–PC5) or made no changes (PC2) in the dynamics motion of BL2 in the first five PCs of active USP14, while decreasing BL1 motion in PC2 but increasing in PC4 (Figure S7). Moreover, a substantial decrease in BL1 dynamics was observed only in the PC2 of active phospho-USP14 (Figure S7), whereas phosphorylated inactive USP14 showed decreased BL1 dynamics in all PCs (PC1, PC4, PC5, and minor in PC3) except PC2 (Figure S6). The porcupine plots of PC4 and PC5 for phosphorylated inactive and active USP14 rendered in Figure 3F,G confirmed the lack of directed motion in the BL1 and BL2 regions compared to Figure 2D. These results collectively suggest that phosphorylation of USP14 between the active and inactive states may modulate the dynamic characteristics of blocking loops, which might be essential for phospho-dependent derepressing activity.

### 3.3. Phosphorylation Affects the Dynamic Interactions in the Inhibitory Network of Blocking Loops

**Figure 4:**
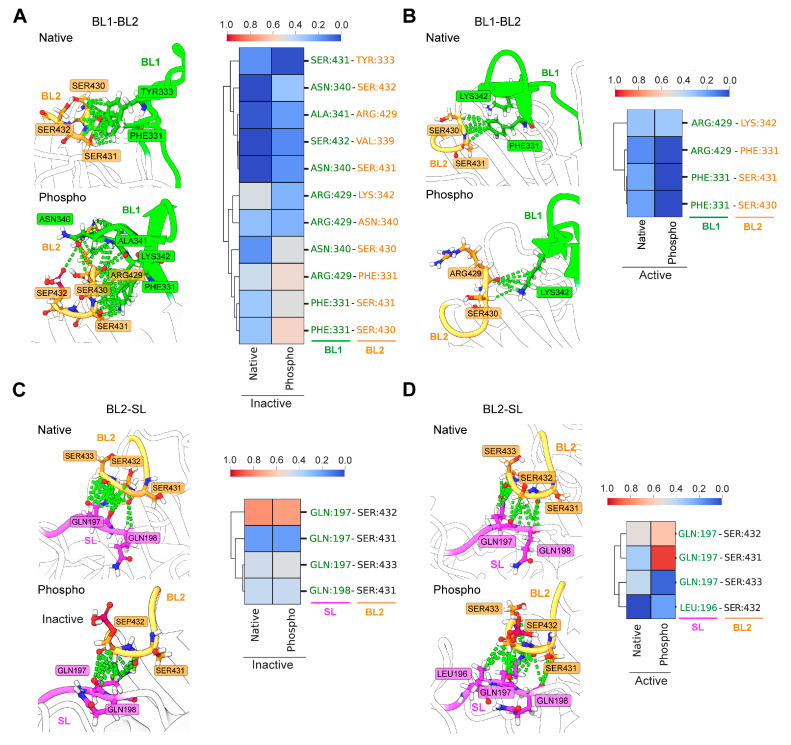
Phosphorylation of Ser432 changes the dynamic interactions in the inhibitory network of blocking loops in USP14. The illustration shows the interloop interactions between BL1 and BL2 in the most populated cluster of native or phosphorylated forms of (**A**) inactive and (**B**) active USP14 trajectories. In each case, the heatmap describes the frequency of contact between the loops during the simulation. The color-coded scale bar (from blue to red) indicates the fraction of contact frequency in the simulation. (**C**,**D**) describes the changes in interloop interaction between the BL2 and SL for inactive and active USP14, respectively. The annotation is the same as for (**A**,**B**).

Inter-residual interactions between the loops determine the rearrangement of BL1 and BL2, directing toward either repression or derepression of USP14 activity [19]. Therefore, it is essential to analyze the shifts in the interactions among the loops due to phosphorylation. Examining the frequency of the non-bonded interactions between BL1 and BL2 in the inactive state (Figure 4A) revealed that phosphorylation increased the interloop interactions, especially by Phe331 and Asn340, which made contact with Arg429, Ser430, and Ser432, suggesting that phosphorylation highly influences the interloop communication of BL1 and BL2. On the other hand, phosphorylation in the active state shows a lower frequency of contact formation between residues of BL1 and BL2 (Figure 4B).

When analyzing the interaction between BL2 and SL, phosphorylation in the inactive state induced only a minor increase in the contact formation between BL2 and SL, where residues in SL, notably Gln197, formed slightly higher interactions with Ser433 in BL2 than their native state (Figure 4C). By contrast, the interloop interactions between BL2 and SL in the active state were dramatically increased by phosphorylation compared to its native form (Figure 4D), where Leu196 and Gln197 from SL maintained substantial interaction with Ser431 and Ser432. These results strongly suggest that phosphorylation on USP14-Ser432 changes the dynamic interactions in the inhibitory network of blocking loops.

### 3.4. Phosphorylation Induces a β-Sheet Structure in the Inactive USP14 BL1 Loop

**Figure 5:**
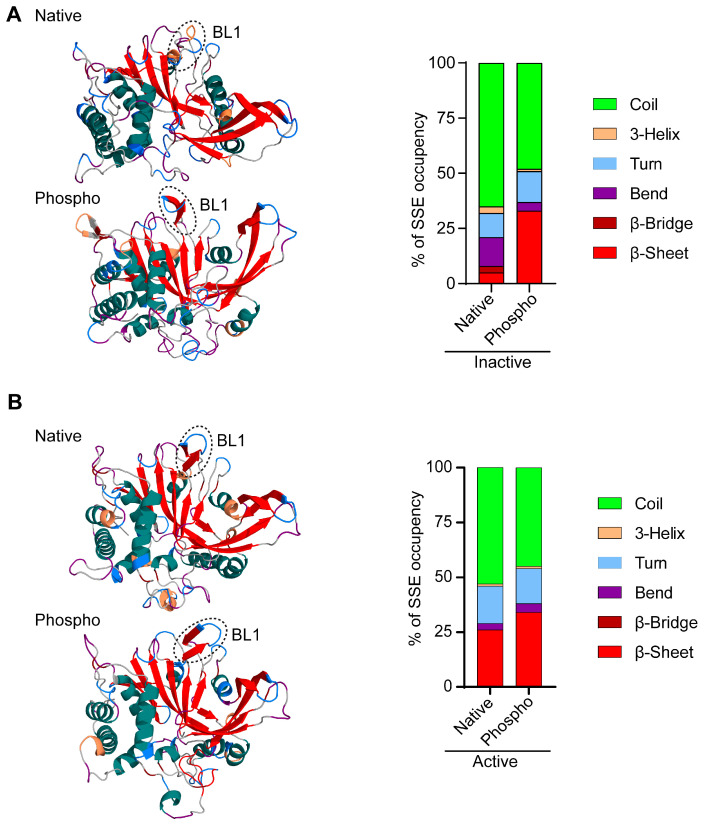
Phosphorylation of Ser432 induces secondary structure formation in the USP14 BL1 loop. (**A**) The most popular structural snapshot obtained from trajectory cluster analysis shows the structural change into a β-sheet within the BL1 loop (marked area) in native and phosphorylated inactive USP14. The panel on the right side shows a stacked bar plot of the secondary structure elements (SSE) occupancy percentage in BL1 classified by the DSSP algorithm. Each color code of the representative structure is given in the key. (**B**) Analysis of secondary structure elements for BL1 in native and phosphorylated active USP14. Annotations are the same as in (**A**). 3-helix, 3_10_-helix.

As PCA analysis evidenced substantial change in conformational dynamics of BL1 by phosphorylation in both inactive and active USP14, we dissected gross variations in the secondary structure of the BL1 loop during the simulation by using the DSSP algorithm [37], which reveals many frames of each residue constituting a 3_10_-helix, α-helix, β-strand, unstructured strand, loop, or bend. Figure 5A shows the representative structures found in the highest population in the MD simulation, where BL1 in the native inactive structure of USP14 does not show any secondary structure formation. However, the phosphorylated USP14 inactive structure strikingly conferred the presence of a β-sheet, which occupies around 33% of the total SSE, substantially higher than the native form of inactive USP14. As such, phosphorylation reduced the propensity for coil, bend, and β-bridge formation in the BL1 loop. Notably, BL1 in native or phosphorylated active USP14 already forms the β-sheet; thus, only minor differences exist in their β-sheet occurrences (Figure 5B).

### 3.5. Phosphorylation Increases the Salt Bridge Interactions between USP14 and Ub

**Figure 6:**
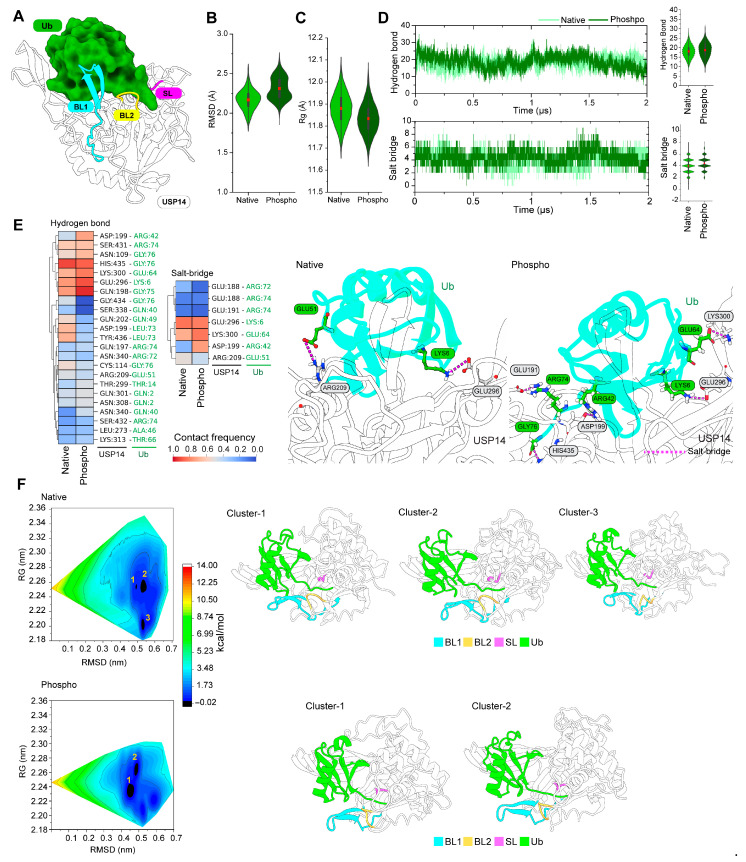
Effect of phosphorylation on the binding of the USP14-Ub complex. (**A**) 3D structural representation of the native USP14-Ub complex highlights the blocking loop conformation in the complex. Violine plots showing conformational changes of Ub in complex with both native and phosphorylated USP14, utilizing RMSD (**B**) and Rg analysis (**C**). (**D**) Line plots show the number of hydrogen bonds (upper panel) and salt bridge interactions (bottom panel) formed between the Ub and native or phosphorylated USP14. The right side of each panel shows the distribution and average contact formation in the violin plot. Inside each violin plot (including **B**–**D**), the red box indicates the mean and the quartiles with whiskers indicating maximum and minimum values. (**E**) Analysis of the hydrogen bond and salt bridge interactions of the USP14-Ub complex. The color-coded heatmaps represent the frequency of hydrogen bonds and salt bridge formation during the simulation. The color-coded scale bar (from blue to red) indicates the fraction of contact frequency in the simulation. The right side of the panel demonstrates the most popular structural snapshot obtained from trajectory cluster analysis of the USP14 complex, which signifies the salt bridge formation (dotted line in pink) for both native and phosphorylated states. (**F**) 2D contour maps showing the free energy landscape (FEL) for the native and phosphorylated USP14-Ub complex. The energy state of the protein conformer is demonstrated by a color-coded map, where the lowest energy minimum is shown in dark blue while red shows a high state. FEL of the native USP14-Ub complex recognized three free energy basins (marked as 1 to 3), while two free energy basins (marked as 1 and 2) were identified in FEL of phospho-USP14 in its Ub bound form. The right side of each plot shows the representative clusters in 3D cartoon models obtained from FEL analysis.

Next, we tried to understand the effect of phosphorylation on the binding of the USP14-Ub complex (Figure 6A). Figure 6B,C demonstrate the stability and flexibility of Ub in the active site of native and phosphorylated USP14 utilizing RMSD and Rg analysis. RMSD analysis showed that Ub in the phosphorylated USP14 is flexible during the simulation, while Rg analysis indicated a more stable geometric conformation of Ub in the phosphorylated USP14 compared to the native USP14. When the intermolecular interactions between Ub and USP14 were analyzed, the phosphorylated USP14 made more hydrogen bonds with Ub than the native one (Figure 6D). Remarkably, salt bridge interactions between Ub and USP14 were dramatically increased by phosphorylation compared to their native form. Heat maps were generated based on the frequency of contact between the Ub and USP14 to identify the critical residues involved in the change of hydrogen bond and salt bridge interactions, as shown in Figure 6E. Residues, including Gln198, Asp199, Glu296, Lys300, Gln301, Asn308, and Asn340 of USP14, were found to maintain high hydrogen bonding with the several residues of Ub, including Gln2, Lys6, Thr14, Gln40, Arg42, Arg74, Gly75, and Gly76, upon phosphorylation. Among these residues, Asp199 and Lys300 maintained a pronounced salt bridge interaction with Arg42 and Glu64, respectively. The visualization of the most populated MD conformer from trajectory clustering analysis also confirmed the difference in salt bridge interactions between the two states. For example, contrary to native USP14, the salt bridge interactions between Arg42 and Asp199 remained sustainable due to phosphorylation.

To reveal the effect of phosphorylation on the conformational dynamics of the USP14-Ub complex, the analysis of the free energy landscape (FEL) for both native and phosphorylated USP14-Ub complexes was further incorporated by considering RMSD and Rg as reaction coordinates 1 and 2, respectively (Figure 6F). By differentiating between the thermodynamic and kinetic properties of the native and phosphorylated USP14-Ub complexes during simulation, FEL provides a more precise representation of the time- and energy-dependent protein conformational space [41]. As shown in the contour map in Figure 6F, black and dark blue regions indicate energetically favorable protein conformations having stable lowest energy states, whereas the native USP14-Ub complex showed three energy-minima basins. In comparison, the phosphorylated complex demonstrated only two major energy-minima basins. In the FEL of the native USP14-Ub complex, most of the conformers were concentrated in cluster 2, followed by 3 and 1. On the other hand, clusters 1 and 2 in the phosphorylated USP14 complex contained the majority of conformers, although they tend to share the same structural characteristics. This observation indicates that phosphorylation affects the binding stability of the complex. Representative conformers from the individual clusters in native and phosphorylated complexes revealed substantial differences in conformational changes in BL1, where BL1 remained closer to the Ub and BL2 in all clusters of the native USP14 complex. In the clusters of phosphorylated USP14, BL1 was found more outward from the USP14 catalytic triads (Figure 6F).

## 4. Discussion

In this study, we employed MD simulations on a microsecond scale to probe the effects of AKT-mediated phosphorylation of the Ser432 residue on the activation of USP14. Although the previous study emphasized the dynamic transition of the BL2 loop for autoinhibitory activity [22], our structural dynamic analysis suggests a different mechanistic insight on USP14 activation associated with BL1 conformational transition and Ub binding.

At the initial point of the study, when we compared the dynamics between active and inactive USP14, we found substantial differences in the conformational dynamics of BL2 between the two states (Figure 2), which explains a possible mechanism for activation, consistent with previous reports [18,22]. Interestingly, when phosphorylation was introduced in the inactive USP14, BL2 conformational dynamics were found to change, but not similar to the ones observed between the inactive and active USP14 (Figure 2C–E), and BL2 seemed to be more stabilized by intramolecular interactions (Figure 3). Based on the interloop interaction analysis, we revealed that phosphorylation increases interloop interaction, especially between the BL2 and BL1 or SL (Figure 4). Analysis of the dynamics of native and phosphorylated USP14 in both active and inactive states evidenced that the BL2 residual interactions with Gln197 of SL and Phe331 of BL1 are critical for the stability of BL2 autoinhibitory conformation and also for the BL1 conformational change. Indeed, the addition of a phosphate group to the serine (Ser432) residue directly contributes to the increase in the interaction of BL2 with Gln197 (SL) and Asn340 (BL1) (Figure 4A,D).

PCA analysis of native and phosphorylated USP14 in inactive and active states revealed the dynamic changes of BL1 in the simulation (Figure 3, Figures S6 and S7). Interestingly, when BL1 was subjected to secondary structure analysis, the phospho-inactive and active USP14 revealed the presence of more β-sheet structures than their native forms (Figure 5), which may serve as a critical mechanism for USP14 activation. In fact, a recent cryo-EM study revealed a crucial mechanism for USP14 activation, where BL1 in USP14 is folded into a β-strand conformation to hold Ub, which is also essential for USP14 interaction with the OB ring of the proteasome [13]. Moreover, our simulation analysis of the USP14-Ub complex showed that phosphorylation substantially induces hydrogen bonding and salt bridge interaction between the Ub and USP14 (Figure 6). Notably, the salt bridge interaction of USP14 with the Arg42 residue of Ub, which was previously identified as critical for USP14 catalytic activity [13], was substantially increased by phosphorylation. Analysis of the non-bonded interaction between BL1 and Ub also suggests a possible mechanism that the conformational transition of BL1 from an open loop to a β-sheet may provide additional surface areas for allowing more interactions with Ub (Figure S8).

Still, questions remain about the contribution of BL2 to USP14 activation during phosphorylation. Phosphorylation changes BL2 flexibility (Figure 3D), which does not lead to open and closing motion but is essential for changing dynamic interactions with other blocking loops (BL1 and SL). These dynamic interactions allow BL1 to adopt the folded structure and shift more outward from the catalytic site to permit Ub access to the catalytic Cys. In the case of BL2 opening conformation in the USP14 active state, the BL2 must be transited from the inactive to the active state of USP14 prior to phosphorylation. This transition could be assisted by the dynamic interactions between USP14 and AKT, where BL2 may undergo a conformational change to access the AKT substrate binding site for phosphorylation. Once a conformational change occurs, phosphorylation at BL2 may sustain this transition and thus persist in an active state. In both cases, BL1 will undergo a conformational change to create an open and broader space to adopt Ub for catalytic activity.

## 5. Conclusions

It is crucial to understand phospho-dependent USP14 activation because abnormal USP14 activity by AKT phosphorylation may promote tumor cell survival and proliferation by deregulating the global protein turnover rate. Using MD simulation, our study presents a novel mechanism of USP14 activation by AKT-mediated Ser432 phosphorylation, which is associated with structural remodeling of BL1, such as by adopting the β-sheet structure, increasing hydrogen bonding and salt-bridge interactions with Ub, and sustaining the BL2 characteristics as an active state. As conformational changes in the blocking loops implicate Ub catalysis, proteasome regulation, and inhibitor binding, the current findings provide additional insights into the mechanism of USP14 phosphorylation and activation, contributing to diverse pathological consequences and also the development of future therapeutics.

## Data Availability

All raw data presented in this paper are available to qualified researchers upon reasonable request.

## Supplementary Materials

The following supporting information can be downloaded at: https://www.mdpi.com/article/10.3390/cells13110955/s1, Table S1. List of simulated systems; Figure S1. The RMSD values from the native (A) and phosphorylated (B) inactive USP14 simulation were calculated from each run of the specific system utilizing the protein c-alpha, respectively; Figure S2. The RMSD values from the native (A) and phosphorylated (B) active USP14 simulation were calculated from each run of the specific system utilizing the protein c-alpha, respectively; Figure S3. The RMSD values from the native (A) and phosphorylated (B) USP14-Ub complex simulation were calculated from each run of the specific system utilizing the protein c-alpha, respectively; Figure S4. The proportion of variance for all principal components (PC) in the inactive (A) and active (B) USP14 simulation in both their native and phospho form; Figure S5. Line plots show the difference between the degree of mobility of inactive and active USP14 captured by PC1 to PC5; Figure S6. Line plots showing the difference between the degree of mobility of native and phospho inactive USP14 captured by PC1 to PC5; Figure S7. Line plots showing the difference between the degree of mobility of native and phospho-active USP14, captured by PC1 to PC5; Figure S8. The total number of non-bonded contact formations between the BL1 of USP14 and Ub during the simulation was compared with the native and phosphorylated forms.
