# Supplementary material for: Structural Dynamics Analysis of USP14 Activation by AKT-Mediated Phosphorylation
Source: Cells. 2024 May 31;13(11):955. doi: 10.3390/cells13110955 (PMC11171753; doi:10.3390/cells13110955)
Supplement: Supplementary file 1 [file cells-13-00955-s001.zip › cells-3001707-supplementary.pdf]

Supplementary File

Table S1. List of simulated systems.

Name of simulated system	Simulation time	Number of Replica	Total Simulation Length
Native Inactive USP14	250 ns	4	1 μs
Phospho Inactive USP14	250 ns	4	1 μs
Native active USP14	200 ns	1	0.95 μs
	250 ns	3	
Phospho active USP14	200 ns	1	0.95 μs
	250 ns	3	
Native USP14-Ub complex	500 ns	4	2 μs
Phospho USP14-Ub complex	500 ns	4	2 μs

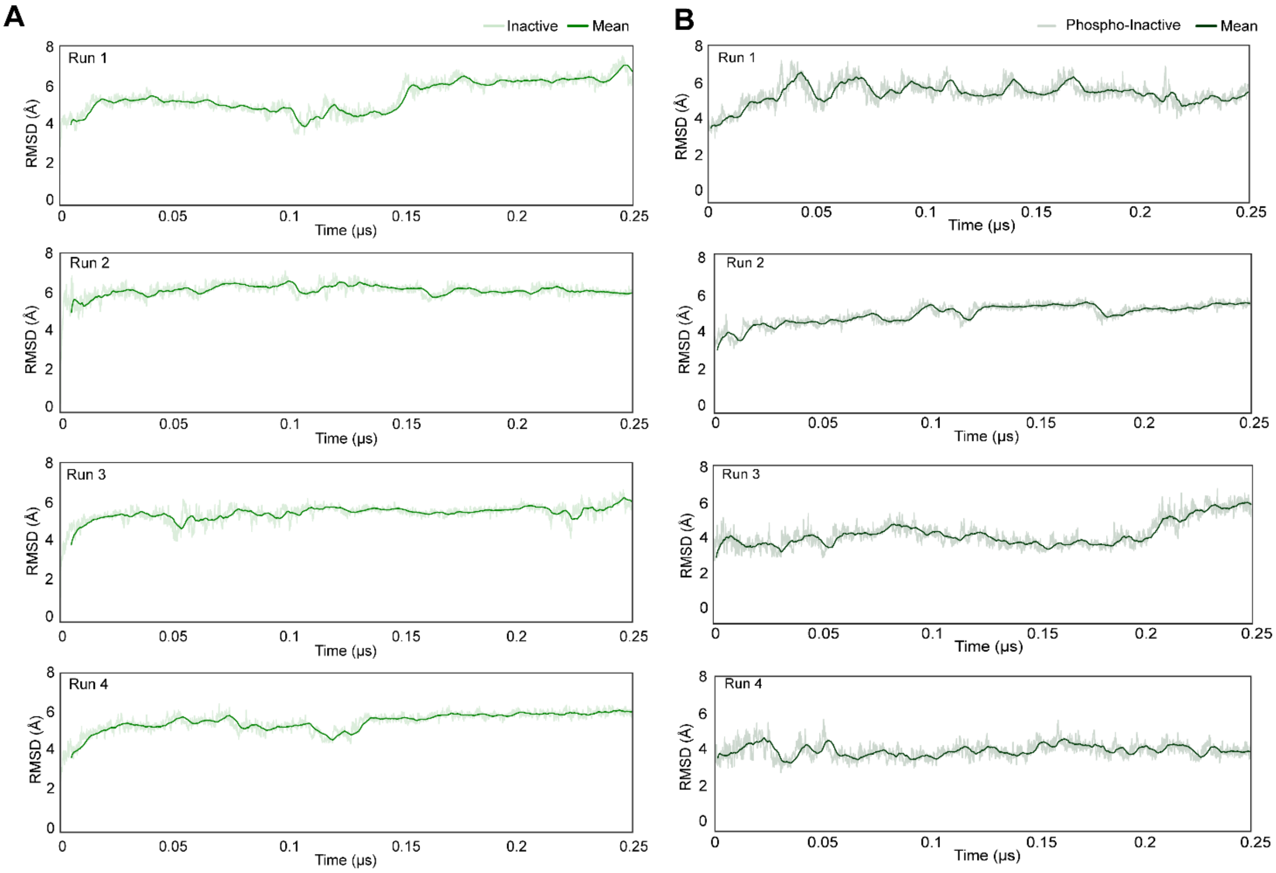

Figure S1. The RMSD values from the native (A) and phosphorylated (B) inactive USP14 simulation were calculated from each run of the specific system utilizing the protein c-alpha, respectively.

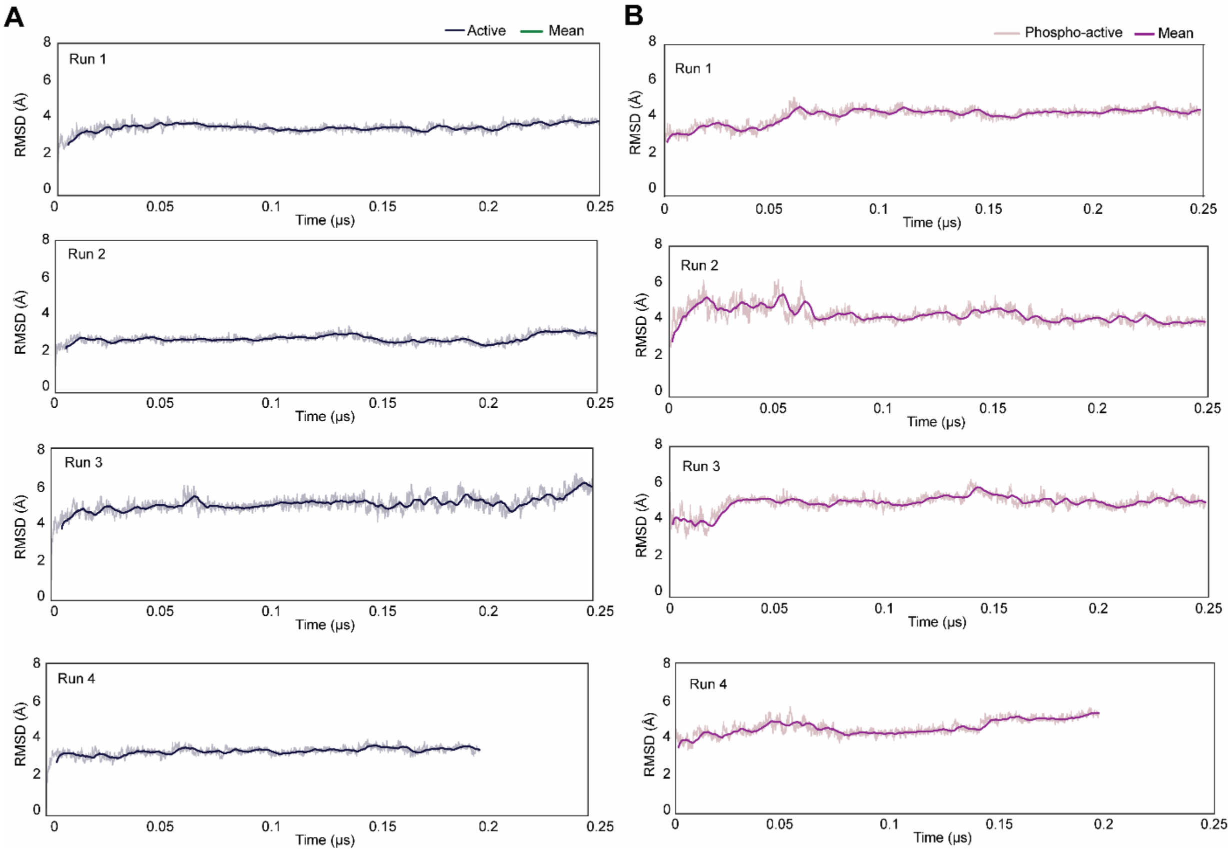

Figure S2. The RMSD values from the native (A) and phosphorylated (B) active USP14 simulation were calculated from each run of the specific system utilizing the protein c-alpha, respectively.

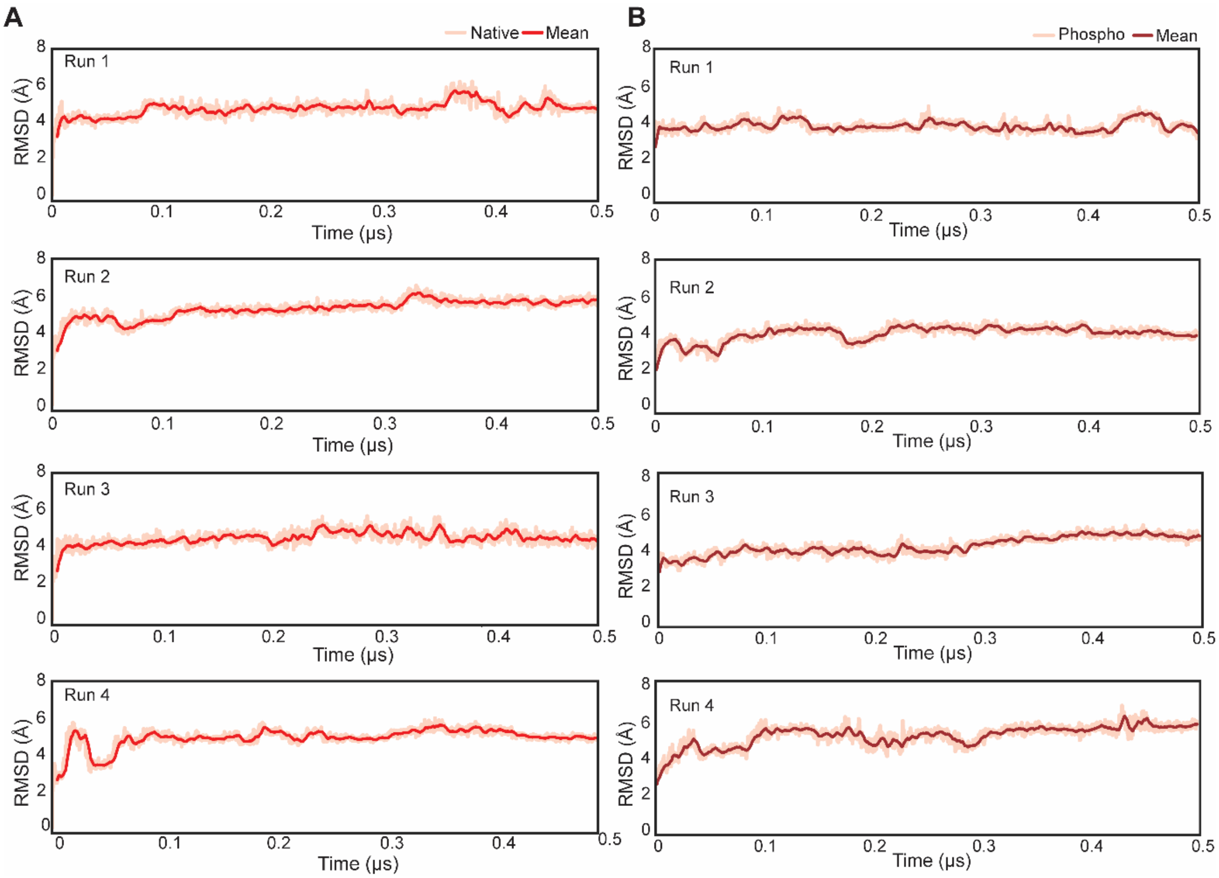

Figure S3. The RMSD values from the native (A) and phosphorylated (B) USP14-Ub complex simulation were calculated from each run of the specific system utilizing the protein c-alpha, respectively.

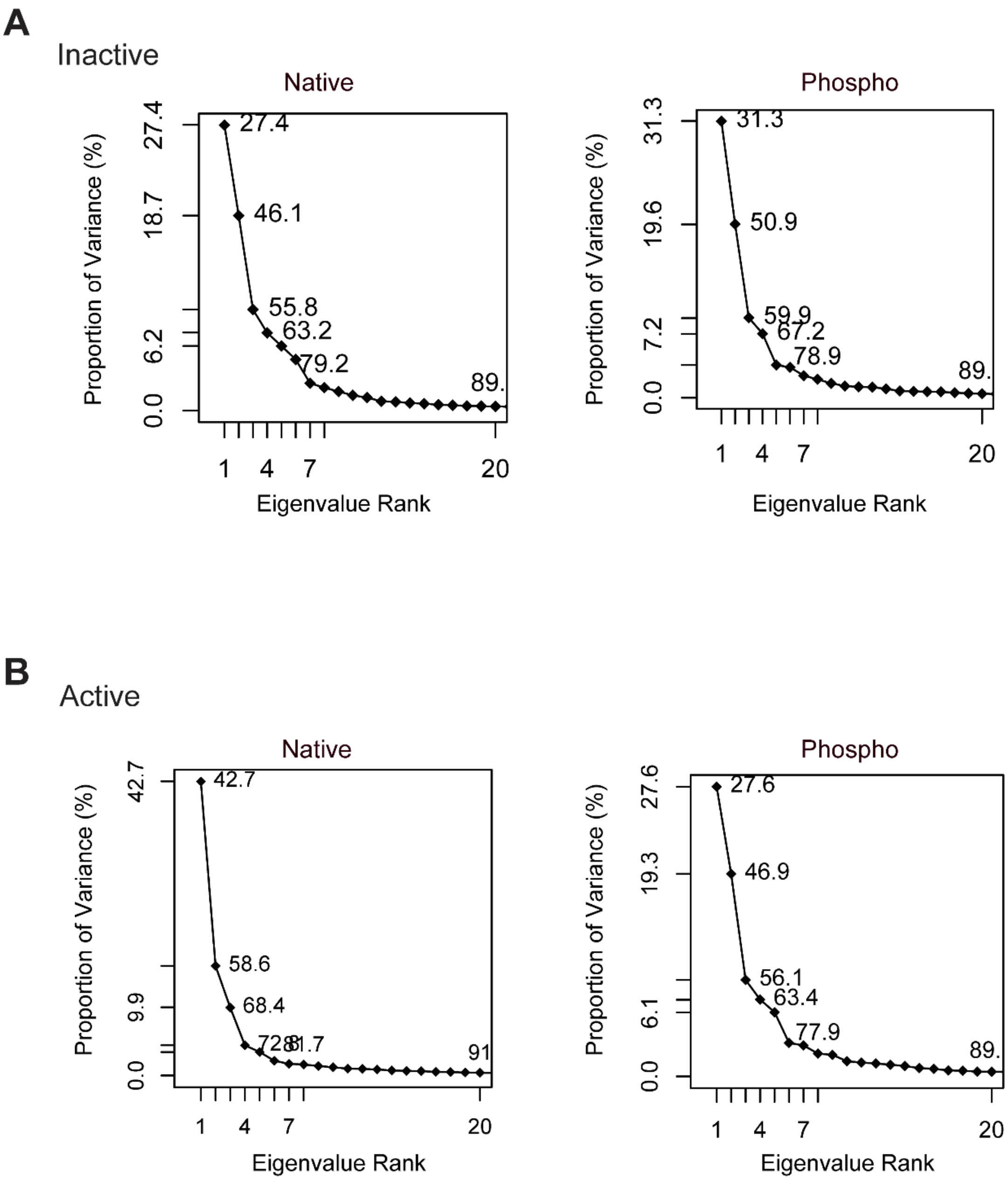

Figure S4. The proportion of variance for all principal components (PC) in inactive (A) and active (B) USP14 simulation in both their native and phospho form.

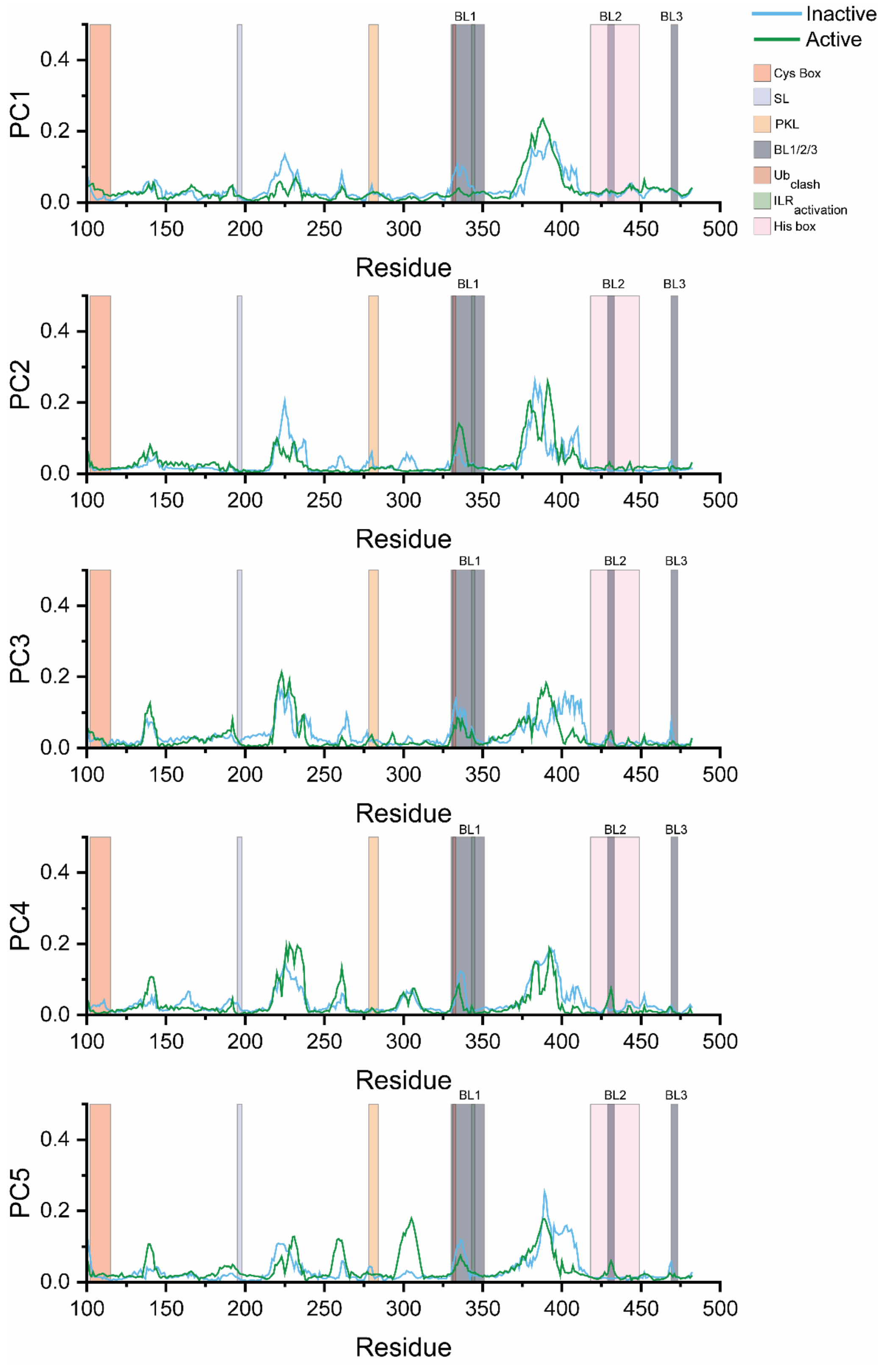

Figure S5. Line plots show the difference between the degree of mobility of inactive and active USP14 captured by PC1 to PC5.

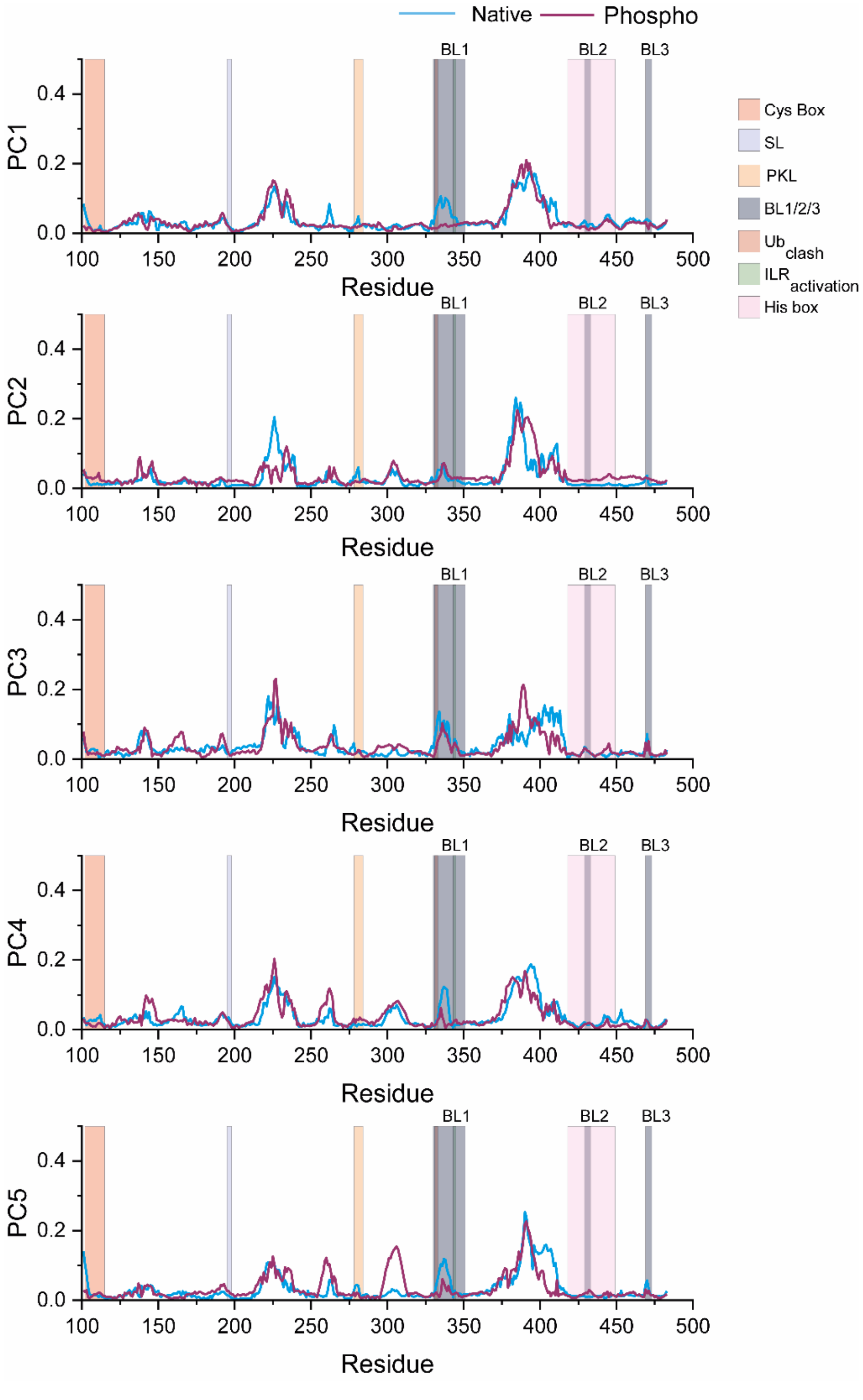

Figure S6. Line plots showing the difference between the degree of mobility of native and phospho inactive USP14, captured by PC1 to PC5.

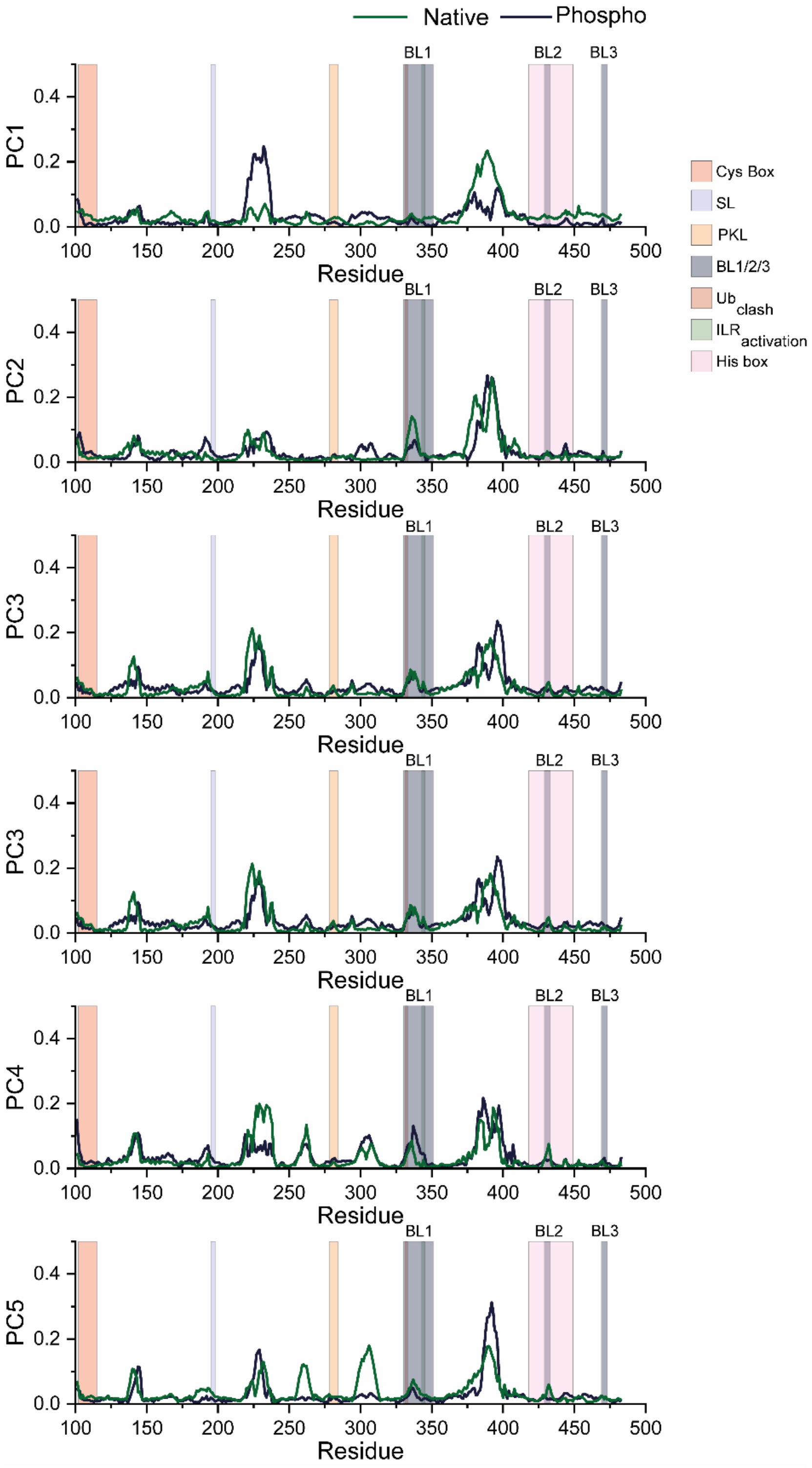

Figure S7. Line plots showing the difference between the degree of mobility of native and phospho-active USP14, captured by PC1 to PC5.

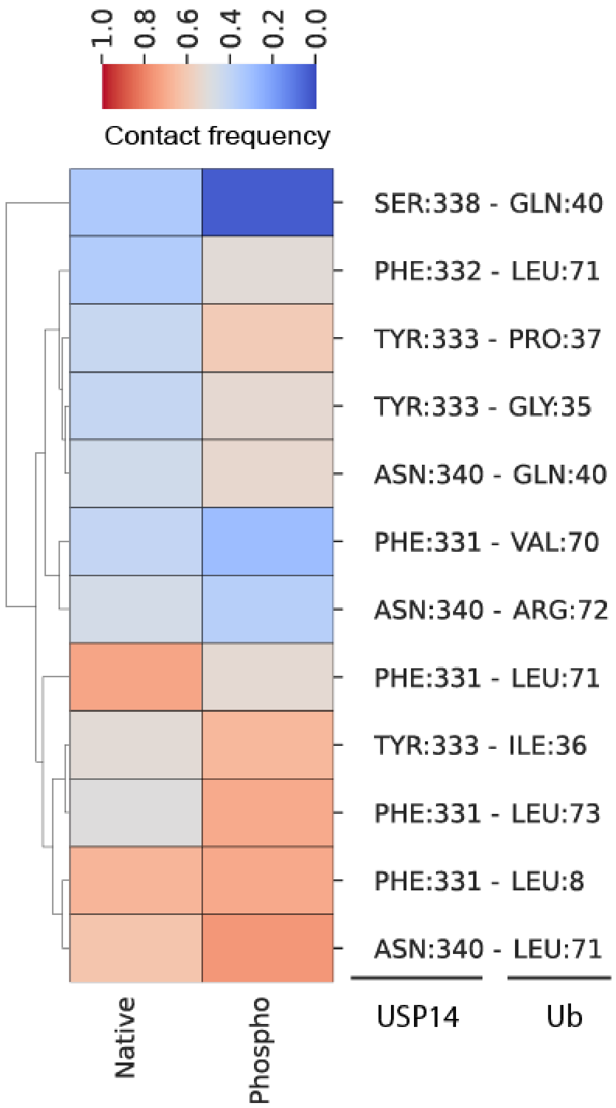

Figure S8. The total number of non-bonded contact formations between the BL1 of USP14 and Ub during the simulation compared with native and phosphorylated form.
